# Case Report: A Novel Homozygous Mutation in *MYF5* Due to Paternal Uniparental Isodisomy of Chromosome 12 in a Case of External Ophthalmoplegia With Rib and Vertebral Anomalies

**DOI:** 10.3389/fgene.2021.780363

**Published:** 2022-02-03

**Authors:** Qianqian Li, Xiaofan Zhu, Chenguang Yu, Lin Shang, Ranran Li, Xia Wang, Yaping Yang, Jingjing Meng, Xiangdong Kong

**Affiliations:** ^1^ Genetics and Prenatal Diagnosis Center, Department of Obstetrics and Gynecology, The First Affiliated Hospital of Zhengzhou University, Zhengzhou, China; ^2^ Key Laboratory of Molecular Biophysics of the Ministry of Education, College of Life Science and Technology and Center for Human Genome Research, Huazhong University of Science and Technology, Wuhan, China; ^3^ Department of Foot and Ankle Surgery, Zhengzhou Orthopedic Hospital, Zhengzhou, China; ^4^ School of Life Science and Technology, Xinxiang Medical University, Xinxiang, China; ^5^ AiLife Diagnostics, Inc., Houston, TX, United States

**Keywords:** external ophthalmoplegia with rib and vertebral anomalies, trio-based exome sequencing, paternal uniparental isodisomy, chromosome 12, the myogenic factor 5 gene, homozygous mutation

## Abstract

External ophthalmoplegia with rib and vertebral anomalies (EORVA) is characterized by congenital nonprogressive external ophthalmoplegia, ptosis, scoliosis, torticollis, vertebral, and rib anomalies, caused by homozygous mutations in the myogenic factor 5 gene (*MYF5*) located on chromosome 12q21.31. Uniparental disomy (UPD) is a rare inheritance of a pair of chromosomes originating from only one parent. This study describes a case of an 8-year-old boy with ptosis, scoliosis, and dysmorphic hypoplastic ribs with fusion anomalies. Trio-based exome sequencing (trio-ES) identified a novel homozygous mutation c.191delC (p.Ala64Valfs*33) in *MYF5* in the proband, with the father being heterozygous and the mother wild-type, as verified by Sanger sequencing. UPD identified from trio-ES variant call format data suggested the possibility of paternal UPD of chromosome 12 (UPD12pat) in the proband, further confirmed to be a complete isodisomy type of UPD by genome-wide single nucleotide polymorphism array. MYF5 was significantly downregulated by 69.14% (**p < 0.01) in HeLa cells transfected with mutant *MYF5* containing c.191delC compared to those transfected with the wild-type *MYF5*, resulting in a truncated protein with a size of ∼20 kDa. In conclusion, this study identified a novel homozygous mutation in *MYF5*, broadening the genetic spectrum of EORVA and further deepening the understanding of this rare disease.

## Introduction

External ophthalmoplegia with rib and vertebral anomalies (EORVA; MIM# 618155), an extremely rare autosomal recessive disorder, is characterized by congenital nonprogressive external ophthalmoplegia and ptosis, with torticollis and scoliosis developing during childhood. In addition, patients may present with hypoplastic or missing ribs with fusion anomalies (Di Gioia et al., 2018). EORVA is caused by homozygous mutations in the myogenic factor 5 gene (*MYF5*; MIM* 159990) located on chromosomal 12q21.31. MYF5, a transcriptional activator encoded by *MYF5*, is a member of the Myc-like basic helix-loop-helix transcription factor family that plays an important role in promoting the transcription of muscle-specific target genes and hence, muscle differentiation (Wang et al., 1996). To date, only two homozygous variations in *MYF5* have been reported (Di Gioia et al., 2018).

Uniparental disomy (UPD), first introduced by Engel (1980), is a non-traditional Mendelian inheritance pattern, in which a pair of chromosomes are inherited from only one parent. It mainly consists of two subtypes: heterodisomy (hUPD), where a pair of non-identical chromosomes are contributed by one parent due to an error during meiosis I, and isodisomy (iUPD), where a chromosome from one parent is duplicated due to an error during meiosis II (Benn, 2021). Recently, Nakka et al. identified 675 instances of UPD across 4,400,363 consented research participants from the personal genetics company 23andMe, Inc., and 431,094 UK Biobank participants, and estimated that the prevalence of UPD on all chromosomes was 1:2000 (0.1%) (Nakka et al., 2019). The clinical consequences of UPD depend on the chromosomes involved, including imprinting diseases, recessive Mendelian diseases, and mosaic aneuploidy associated with diseases (Scuffins et al., 2021).

In this study, an 8-year-old boy with ptosis, scoliosis, and dysmorphic hypoplastic ribs with fusion anomalies was identified with a novel homozygous variation in *MYF5* due to paternal iUPD of chromosome 12 (iUPD12pat).

### Case Presentation

**FIGURE 1:**
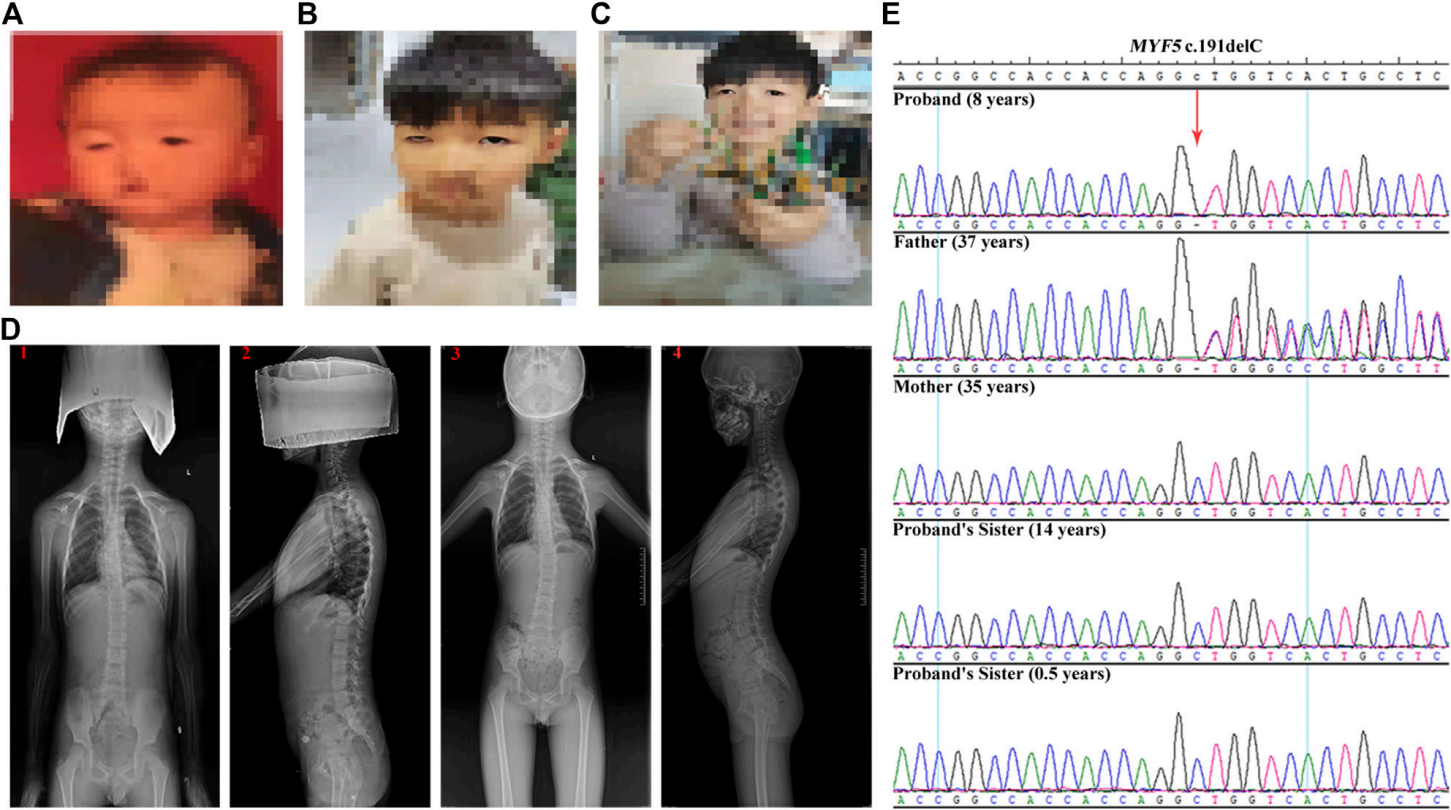
Clinical features of the proband and Sanger sequencing results of the family. **(A–C)** The ptosis phenotype at birth, at the age of 6 years before the reconstructive surgery, and at the age of 6 years after the reconstructive surgery, respectively, showing the improvement in ptosis symptom for the proband. **(D)** DR images acquired at the age of 7 years (1 and 2) and at the age of 8 years (3 and 4), showing the significant aggravation of the scoliosis condition for the proband. **(E)** Sanger sequencing results of c.191delC for the family. The proband is homozygous. The father is heterozygous, whereas the mother and the two sisters are wild-type.

**FIGURE 2:**
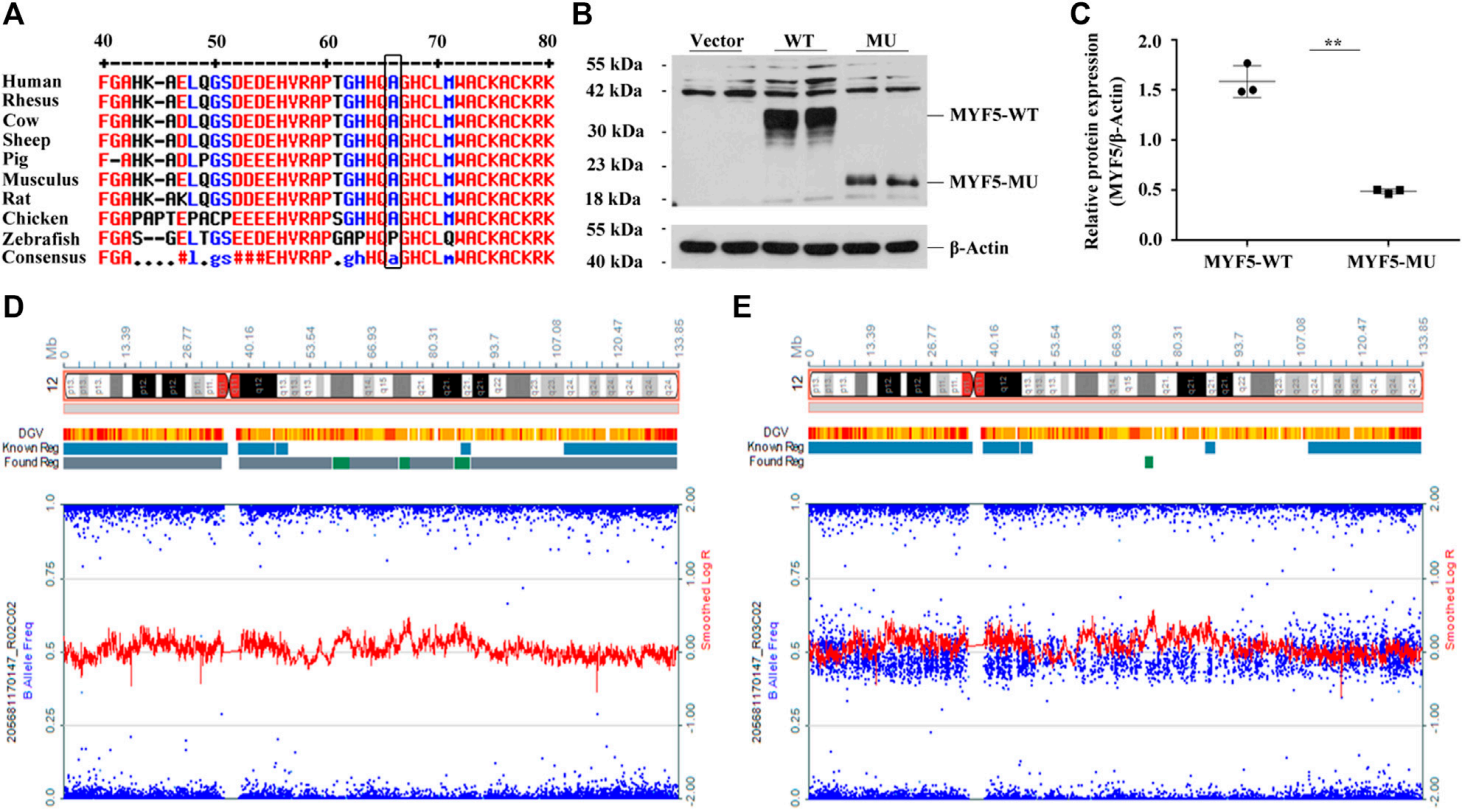
Functional study of c.191delC and genome-wide SNP array results of the proband and the father. **(A)** p.Ala64 is highly conservative across different species. **(B)**, **(C)** Western blotting indicated the significant downregulation of MYF5 by 69.14% (**p < 0.01) in cells transfected with p3×FLAG-CMV-10-*MYF5*-MU compared to those transfected with p3×FLAG-CMV-10-*MYF5*-WT, resulting in a truncated protein with a size of ∼20 kDa. Vector: p3×FLAG-CMV-10. **(D)**, **(E)** Genome-wide SNP array results of the proband and the father, respectively (only chromosome 12 shown).

The proband was an 8-year-old boy with ptosis since birth (Figure 1A). At the age of 1 year, the proband was diagnosed with ptosis by magnetic resonance imaging of the ocular motor nerve. At the age of 6 years, the proband underwent reconstructive surgery, and the symptom of ptosis was improved (Figure 1B, before surgery; Figure 1C, after surgery). And at the age of 6 years, the proband was further diagnosed with scoliosis by computed tomography at a local hospital (data not shown) and further diagnosed with scoliosis and dysmorphic hypoplastic ribs with fusion anomalies, however, with no vertebral anomalies, by digital radiography (DR) at Zhengzhou Orthopedic Hospital at the age of 7 years (Figure 1D, **1** and **2**). At the age of 8 years, the DR images suggested a significant aggravation of scoliosis for the proband (Figure 1D, **3** and **4**).

## Methods

### Ethics Approval

The family provided written informed consent to carry out this study. This study was approved by the appropriate local institutional review board on human subject research at the First Affiliated Hospital of Zhengzhou University.

### Quantitative Fluorescent Polymerase Chain Reaction

Genomic DNA was extracted from 500 μL of peripheral blood using the Lab-Aid Nucleic Acid (DNA) Isolation Kit (Zeesan, Xiamen, China). The genetic relationship of the proband and the parents was confirmed by QF-PCR using the Goldeneye™ DNA ID System 20A Kit (Peoplespot, Beijing, China).

### Trio-Based Exome Sequencing

The methods of experiment and data analysis used for trio-ES have been described in detail in our previous study (Li et al., 2021). The candidate mutation in *MYF5* was verified by Sanger sequencing using the primer pairs provided in Supplementary Table S1.

### Bioinformatics Analysis

The conservation analysis was performed using the MultAlin tool (http://multalin.toulouse.inra.fr/multalin/multalin.html). The variant frequency in different populations was obtained from 1000G (https://www.internationalgenome.org/), gnomAD (http://gnomad-sg.org/), and ExAC databases (http://exac.broadinstitute.org). SIFT (http://provean.jcvi.org/protein_batch_submit.php?species=human), PolyPhen2 (http://genetics. bwh.harvard.edu/pph2/index.shtml), and MutationTaster (https://www.mutationtaster.org/) were used to predict the deleterious effects of the variant on the protein structure and function.

UPD was identified online through AilisNGS^®^ (“https://www.ailifeus.com/#/products/ngs”. Houston, USA) from the trio-ES variant call format (vcf) data as per the method previously described (King et al., 2014).

### Genome-Wide Single Nucleotide Polymorphism Array

Genome-wide SNP array, including fragmentation, labelling, and hybridization, was carried out using DNA samples from the proband and the father using Illumina HumanCytoSNP-12 v2.1 BeadChip (California, USA) on Illumina iScan. The methods of experiment and data analysis have been previously described (Bruno et al., 2011).

### Cell Line, Plasmids, and Transfection

HeLa cells (human epithelial cervix carcinoma cells, American Type Culture Collection) were cultured in Dulbecco’s modified Eagle’s medium (Gibco Life Technologies, Gaithersburg, MD, USA) supplemented with 10% fetal bovine serum (Gibco Life Technologies) and 5% CO_2_ at 37°C.

The full-length coding region of *MYF5* was sub-cloned into p3×FLAG-CMV-10 (p3×FLAG-CMV-10-*MYF5*-WT), whereas the mutant plasmid was constructed using the PCR-based site-directed mutagenesis (p3×FLAG-CMV-10-*MYF5*-MU) using the primers given in Supplementary Table S1.

HeLa cells were cultured for 24 h and then transfected with 2 μg of p3×FLAG-CMV-10-*MYF5*-WT, p3×FLAG-CMV-10-*MYF5*-MU, or p3×FLAG-CMV-10.

### Western Blotting

Following 48 h incubation with 5% CO_2_ at 37°C, the cells were collected and lysed in lysis buffer. Then, the cell lysate was separated using 10% sodium dodecyl sulfate-polyacrylamide gel electrophoresis. After blocking with 5% skimmed milk powder in 1 × TBST buffer (10 mM Tris, 150 mM NaCl, and 0.05% Tween-20, pH7.5), the membrane was incubated with the primary antibody (anti-DDDDK tag, M185-3L, Medical & Biological Laboratories Co., Ltd, Nagoya, Japan) at 4°C overnight. Following washing with 1 × TBST buffer, the membrane was incubated with the secondary antibody (BL001A, Biosharp, Anhui, China). β-Actin (GB11001, Servicebio, Wuhan, China) was used as a loading control.

### Statistical Analysis

Data are presented as the mean ± SEM. Statistical analysis was performed using GraphPad Prism 6. Student’s two-tailed *t*-test was used for between-group comparisons. Statistically significant differences were considered at *p* < 0.05 (**p < 0.01).

## Results

The genetic relationship between the proband and the parents was confirmed by QF-PCR (Supplementary Table S2 and Supplementary Figure S1). The quality control of the trio-ES data is summarized in Supplementary Table S3. After filtering, only the homozygous candidate variant c.191delC (p.Ala64Valfs*33) in *MYF5* (NM_005593.3) was identified in the proband (reference allele/alternative allele, ref/alt: 0/78), with the father heterozygous (ref/alt: 40/32) and the mother wild-type (Supplementary Figures S2–4), as confirmed by Sanger sequencing (Figure 1E).

Online prediction using MultAlin indicated that p.Ala64 was highly conservative across different species (Figure 2A, black box), and c.191delC was absent in 1000G, ExAC, and gnomAD databases (Supplementary Table S4). UPD identified based on the trio-ES vcf data suggested the possibility of UPD12pat in the proband (Supplementary Figure S5).

For the proband, log R ratio and B allelic frequency confirmed that UPD was a complete paternal UPD12 spanning 12p13.33-q24.33, named arr [GRCh37/hg19]12p13.33q24.33 (413,635-133,272,968)×2 hmz according to the International System for Human Cytogenomic Nomenclature (ISCN 2016), including the paternal mutant allele of *MYF5* (Figure 2D, the proband; **2E**, the father). All SNPs are listed in Supplementary Excel S1 (205681170147_R02C02, the proband; 205681170147_R03C02, the father).

Western blotting confirmed the significant downregulation of MYF5 by 69.14% (**p < 0.01) in cells transfected with p3×FLAG-CMV-10-*MYF5*-MU compared to those transfected with p3×FLAG-CMV-10-*MYF5*-WT, resulting in a truncated protein with a size of ∼20 kDa (Figures 2B,C). These findings suggested that the mutation of c.191delC drastically affects the biological function of MYF5.

## Discussion

External ophthalmoplegia with rib and vertebral anomalies is a rare autosomal recessive disorder characterized by congenital ophthalmoplegia with scoliosis and vertebral and rib anomalies, with only five individuals (three families) reported to date (Di Gioia et al., 2018). In this study, we report a novel homozygous variant in *MYF5* (c.191delC) due to iUPD.

The proband in this study was an 8-year-old boy diagnosed with ptosis, scoliosis, and dysmorphic hypoplastic ribs with fusion anomalies. To the best of our knowledge, individuals with EORVA reported previously (Traboulsi et al., 2000; Di Gioia et al., 2018) and the patient in this study had similar phenotypes regarding external ophthalmoplegia, rib, and vertebral anomalies. In most cases, unilateral or bilateral ptosis was present, including our current case. Additionally, delayed motor development can occur, which was previously reported in one EORVA case but not observed in our case. Cognition was normal in all cases.

For the proband, trio-ES suggested a novel homozygous variant c.191delC in exon 1 of *MYF5*. This variant was heterozygous in the father and absent in the mother (Supplementary Figures S2–4), as further confirmed by Sanger sequencing (Figure 1E). To identify this non-Mendelian inheritance, UPD was analyzed based on the trio-ES vcf data. The result indicated that the homozygous state of c.191delC in the proband may be presumably attributed to UPD12pat (Supplementary Figure S5A), which was finally confirmed to be a complete iUPD by genome-wide SNP array (Figures 2D,E and Supplementary Excel S1).

The novel variant c.191delC results in p.Ala64Valfs*33, causing a frameshift mutation and premature termination of translation at codon 96. To our knowledge, this is the third pathogenic homozygous mutation reported in EORVA patients and the first mutation caused by UPD in *MYF5*. The biological function of MYF5 was severely affected because of the mutant-type (Figures 2B,C). Therefore, regular clinical observations need to be conducted on the proband to evaluate if the clinical symptoms of EOVAR will worsen in the future, though undergoing reconstructive surgery for ptosis.

UPD is the inheritance in which both homologous chromosomes are from one parent with no representative copy from the other, occurring as iUPD, hUPD, a combination of both, or only chromosome segment(s) (Schroeder et al., 2014). In 2020, a UPD study of trios in a clinical cohort of suspected genetic diseases revealed that the incidence of UPD was about 2:1,000 (0.2%) (Yauy et al., 2020). Later in 2021, in a population of 32,067 clinical exome trios, 16 cases were related to a positive test result through homozygous sequence variations (Scuffins et al., 2021).

Complete iUPD, an underestimated cause of recessive Mendelian disorders, is the inheritance of two identical chromosomes that are the same at all polymorphic sites along their length (Middleton et al., 2006), and is the predominant UPD type observed in the largest chromosomes (Del Gaudio et al., 2020). Roberts et al. identified a homozygous nonsense mutation c.1618A > T (p.Lys540*) in *CD45* (NM_002838) in a severe combined immunodeficiency patient caused by maternal iUPD1 (iUPD1mat) (Roberts et al., 2012). Chen et al. represented a case of epileptic disorder associated with a novel mutation c.2873_c.2874delCT (p.Thr958Thrfs*17) in homozygosity in *CNTN2* (NM_005076) due to UPD1mat (Chen et al., 2021). Several autosomal recessive disorders have been reported to be owing to the homozygous mutations on chromosome 2 disclosed by iUPD2pat (Kantarci et al., 2008; Dasi et al., 2016; Hara-Isono et al., 2021). Sasaki et al. reported a 3M syndrome patient with a homozygous mutation c.2975G > C (p.Arg992Pro) in *CUL7* (NM_014780) attributed to iUPD6mat (Sasaki et al., 2011). Cho et al. unmasked a homozygous mutation c.1120C > G (p.Tyr400*) in *SUOX* (NC_000012.11) in a patient with isolated sulfite oxidase deficiency resulting from UPD12pat (Cho et al., 2013). Wiszniewski et al. revealed a homozygous mutation c.1148delC (p.Thr383Ilefs*13) in *CNGB3* (NM_019098) in a case with achromatopsia associated with UPD14mat (Wiszniewski et al., 2007). SoehnSoehn et al. described four novel homozygous mutations in *FA2H* (NM_024306) in four unrelated families with spastic paraplegia type 35 because of UPD16 (SoehnSoehn et al., 2016).

In conclusion, to the best of our knowledge, for the first time, we identified a novel homozygous mutation in *MYF5* due to iUPD12pat in a case of EORVA, a rare event and enriching *MYF5* gene variation spectrum, further suggesting that UPD of any chromosome is associated with an increased risk of recessive disease because it may affect children when only one parent is a carrier of a pathogenic variant. Additionally, the results re-emphasized the clinical importance of UPD in case the results are inconsistent with recessive inheritance.

## Data Availability

The datasets for this article are not publicly available due to concerns regarding participant/patient anonymity. Requests to access the datasets should be directed to the corresponding authors.
