# Supplementary material for: Case Report: A Novel Homozygous Mutation in MYF5 Due to Paternal Uniparental Isodisomy of Chromosome 12 in a Case of External Ophthalmoplegia With Rib and Vertebral Anomalies
Source: Front Genet. 2022 Feb 3;12:780363. doi: 10.3389/fgene.2021.780363 (PMC8851471; doi:10.3389/fgene.2021.780363)
Supplement: Supplementary file 1 [file DataSheet1.zip › Supplementary Materials/Supplementary Tables and Figures-2021-12-13.docx]

**Supplementary Table 1.** Primer sequences for Sanger sequencing and the mutant type plasmid construction.

| **Primer name** | **Primer sequence (Forward)** | **Primer sequence (Reverse)** |
| --- | --- | --- |
| *MYF5*-Sequence | 5′-ACCGGAGCGACAGACTAG-3′ | 5′-GGGGTTGGTCGTGGTAC-3′ |
| p3×FLAG-CMV-10-*MYF5*-MU | 5′-CGGCCACCACCAGGTGGTCACTGCCTC-3′ | 5′-CGGCCACCACCAGGTGGTCACTGCCTC-3′ |

**Supplementary Table 2.** STR loci of the proband and the parents.

| **STR loci** | **Mother** | | **Proband** | | **Father** | | **Father gene 1** | **Father gene 2** | **Calculation formula** | **PI** |
| --- | --- | --- | --- | --- | --- | --- | --- | --- | --- | --- |
| D19S433 | 14 | 14 | 13 | 14 | 13 | 16.2 | 0.2313 |  | 1/(2*p) | 2.1617 |
| D5S818 | 12 | 12 | 12 | 12 | 10 | 12 | 0.2406 |  | 1/(2*p) | 2.0781 |
| D21S11 | 29 | 33.2 | 29 | 33.2 | 29 | 29 | 0.2571 | 0.0443 | 1/(p+q) | 3.3179 |
| D18S51 | 13 | 15 | 13 | 14 | 13 | 14 | 0.2160 |  | 1/(2*p) | 2.3148 |
| D6S1043 | 14 | 20 | 13 | 20 | 13 | 19 | 0.1328 |  | 1/(2*p) | 3.7651 |
| D3S1358 | 17 | 17 | 14 | 17 | 14 | 18 | 0.0473 |  | 1/(2*p) | 10.5708 |
| D13S317 | 9 | 11 | 9 | 11 | 9 | 11 | 0.1318 | 0.2368 | 1/(p+q) | 2.7130 |
| D7S820 | 11 | 11 | 11 | 12 | 12 | 12 | 0.2453 |  | 1/p | 4.0766 |
| D16S539 | 9 | 10 | 10 | 12 | 10 | 12 | 0.2058 |  | 1/(2*p) | 2.4295 |
| CSF1PO | 10 | 12 | 12 | 12 | 10 | 12 | 0.3686 |  | 1/(2*p) | 1.3565 |
| Penta D | 11 | 13 | 8 | 11 | 8 | 11 | 0.0691 |  | 1/(2*p) | 7.2359 |
| AMEL | 0 | 0 | 0 | 1 | 0 | 1 |  |  |  |  |
| **vWA** | **18** | **19** | **21** | **21** | **14** | **21** |  | **0.0000** |  | **1.0000** |
| D8S1179 | 12 | 15 | 15 | 16 | 13 | 16 | 0.0737 |  | 1/(2*p) | 6.7843 |
| TPOX | 8 | 11 | 8 | 8 | 8 | 11 | 0.5136 |  | 1/(2*p) | 0.9735 |
| Penta E | 18 | 20 | 17 | 18 | 14 | 17 | 0.0581 |  | 1/(2*p) | 8.6059 |
| TH01 | 7 | 9 | 7 | 9 | 9 | 9 | 0.2664 | 0.5215 | 1/(p+q) | 1.2692 |
| **D12S391** | **19** | **25** | **24** | **24** | **20** | **24** |  | **0.0000** |  | **1.0000** |
| D2S1338 | 18 | 24 | 18 | 20 | 19 | 20 | 0.1219 |  | 1/(2*p) | 4.1017 |
| FGA | 21 | 25 | 21 | 22 | 22 | 22 | 0.1866 |  | 1/p | 5.3591 |
|  |  |  |  |  |  |  |  | PI (Accumulation) 574282884.0804 | | |
|  |  |  |  |  |  |  |  | RCP 0.9999999983 | | |

**Supplementary Table 3.** Quality control of the trio-ES data.

| **20×Coverage (%)** | | | **Data size (Mb)** | | | **Capture efficiency (%)** | | | **Duplication rate (%)** | | | **Ave. sequencing depth (×)** | | |
| --- | --- | --- | --- | --- | --- | --- | --- | --- | --- | --- | --- | --- | --- | --- |
| Proband | Mother | Father | Proband | Mother | Father | Proband | Mother | Father | Proband | Mother | Father | Proband | Mother | Father |
| 95.47 | 95.01 | 95.2 | 10987 | 10200 | 10261 | 76.31 | 76.6 | 76.38 | 0.123 | 0.131 | 0.118 | 126.0 | 118.17 | 117.46 |

**Supplementary Table 4.** Bioinformatic analysis of c.191delC.

| **Gene**  **Name** | **Location**  **Exon** | **Mutation**  **Type** | **Transcript ID** | **Mutation Site** | **SIFT** | **PolyPhen2** | **Mutation Taster** | **1000G** | **ExAC** | **gnomAD** |
| --- | --- | --- | --- | --- | --- | --- | --- | --- | --- | --- |
| *MYF5* | chr12:81111032  Exon 1 | Frameshift  mutation | ENST00000228644 | c.191delC  p.Ala64Valfs*33 | - | - | 1 | - | - | - |

SIFT, predicts whether an amino acid substitution affects protein function. SIFT can be applied to naturally occurring nonsynonymous polymorphisms or laboratory-induced missense mutations.

PolyPhen2, predicts the effect of an amino acid substitution on the structure and function of a protein using sequence homology, Pfam annotations, 3D structures from PDB where available, and a number of other databases and tools (including DSSP, ncoils etc.).

Mutation Taster, predicts the functional consequences of not only amino acid substitutions but also intronic and synonymous alterations, short insertion and/or deletion (indel) mutations and variants spanning intron-exon borders.

**Supplementary Figure 1.** QF-PCR results of the proband and the parents.

**
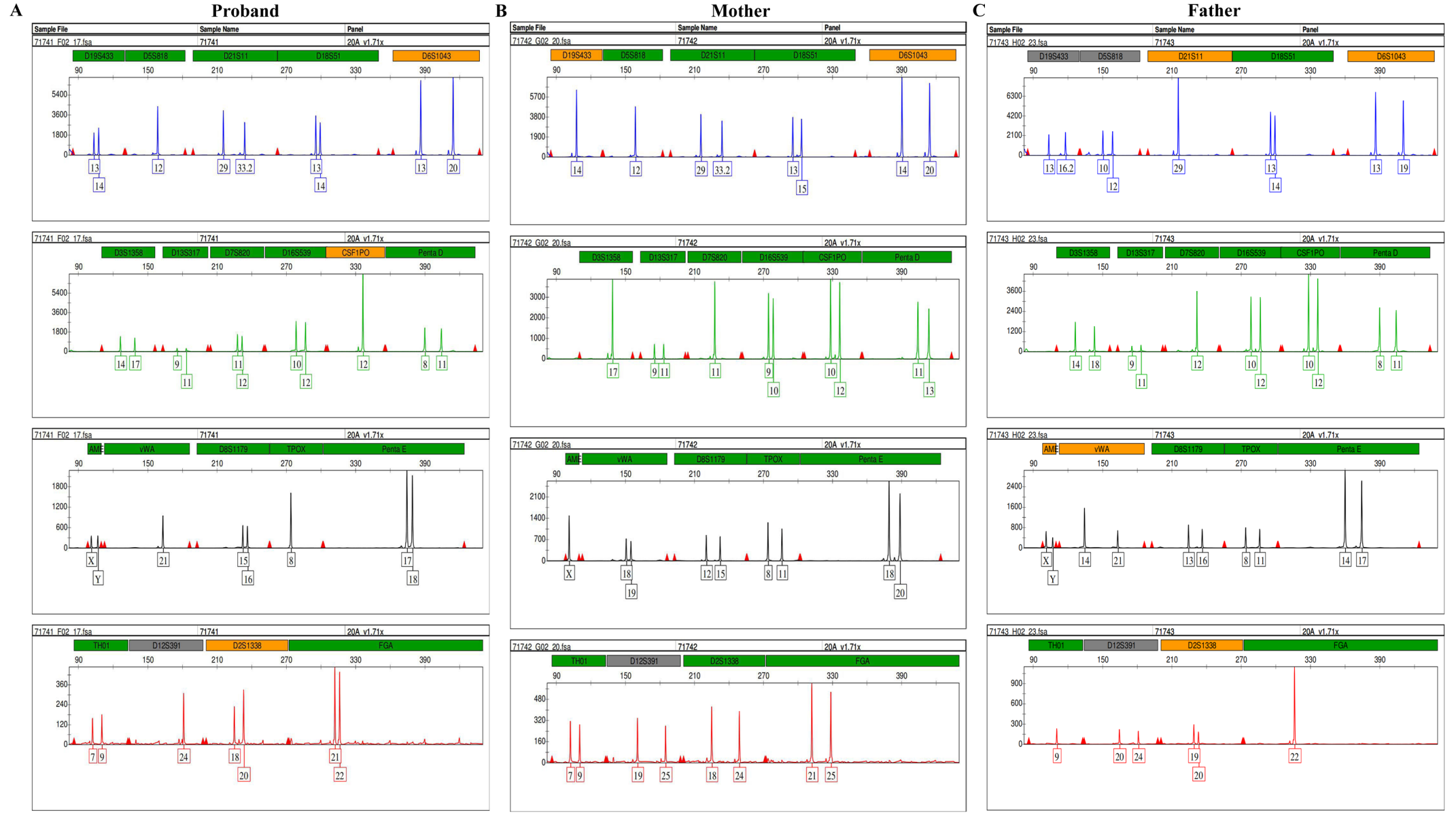
**

**Supplementary Figure 2.** Screenshot of the exome alignment of c.191delC in the proband.

**
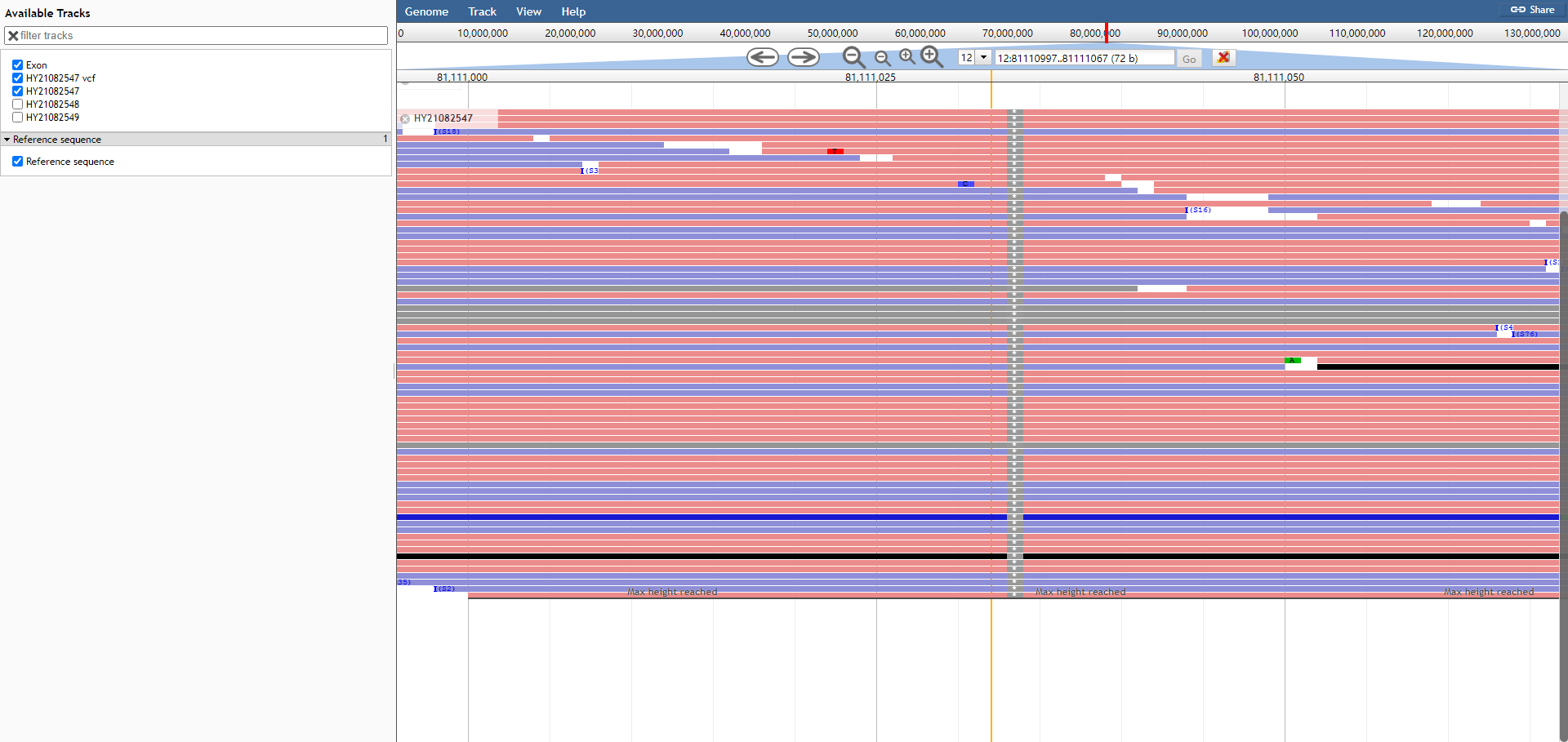
**

**Supplementary Figure 3.** Screenshot of the exome alignment of c.191delC in the father.


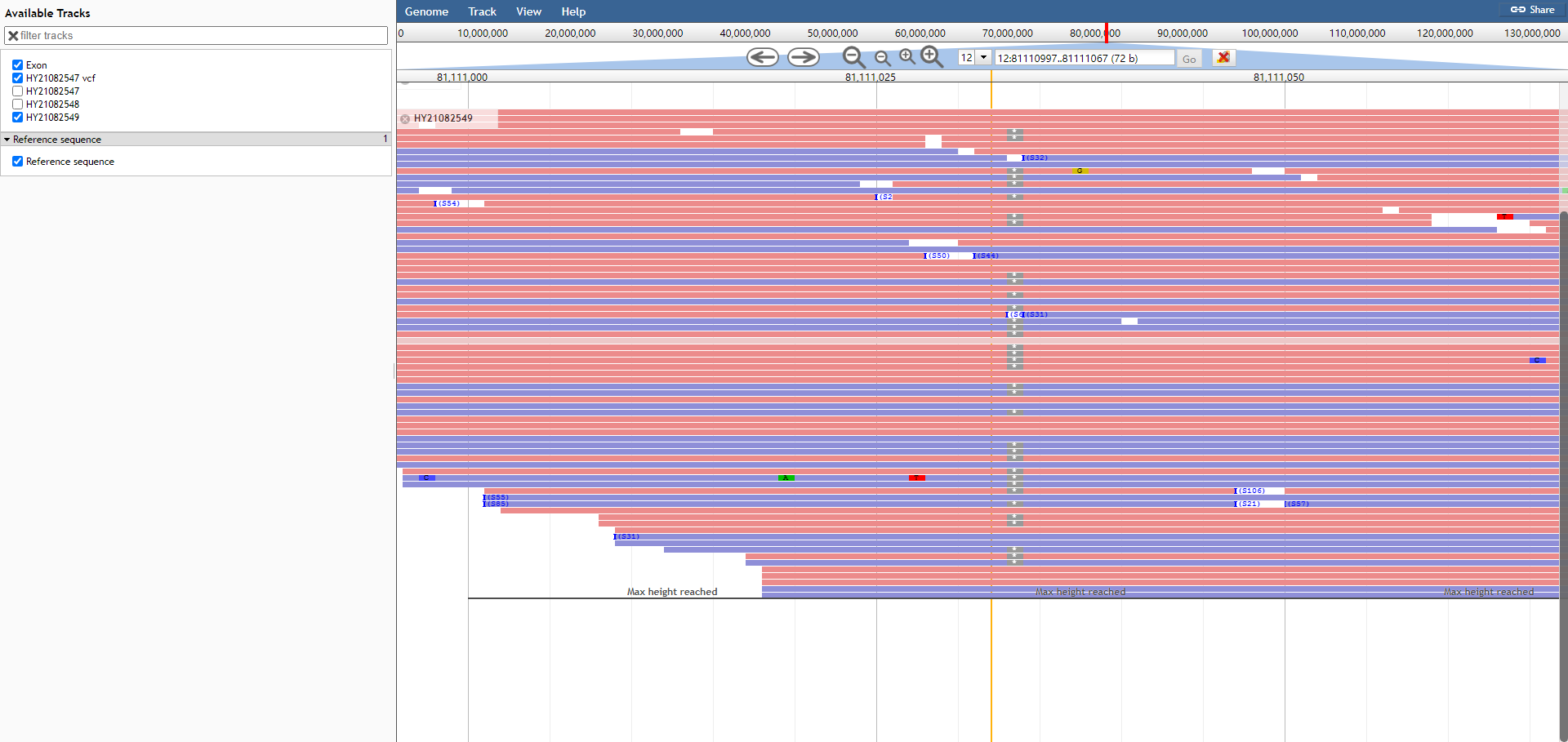


**Supplementary Figure 4.** Screenshot of the exome alignment of c.191delC in the mother.

**
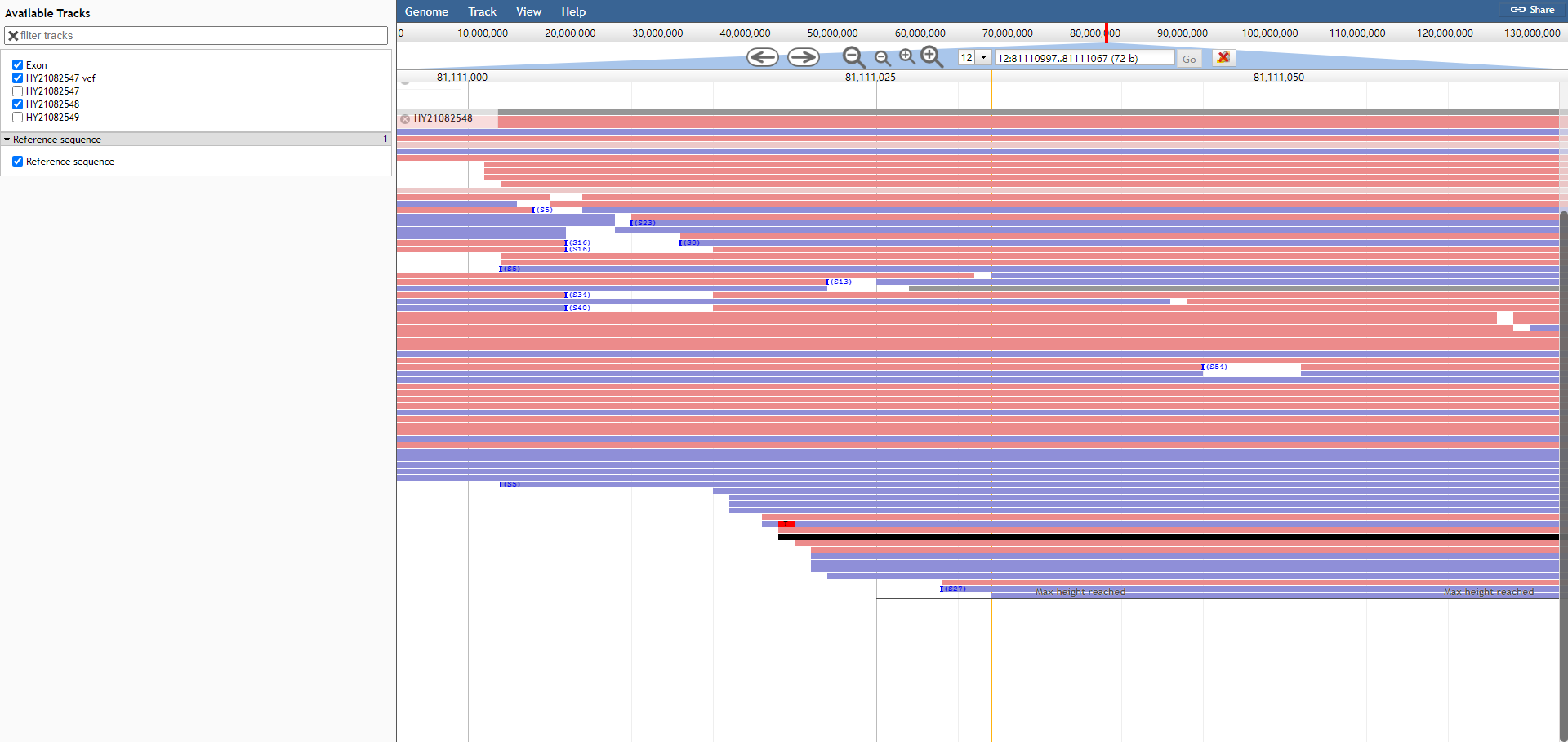
**

**Supplementary Figure 5.** UPD identified from the trio-ES vcf data (A, the proband; B, the father).

**
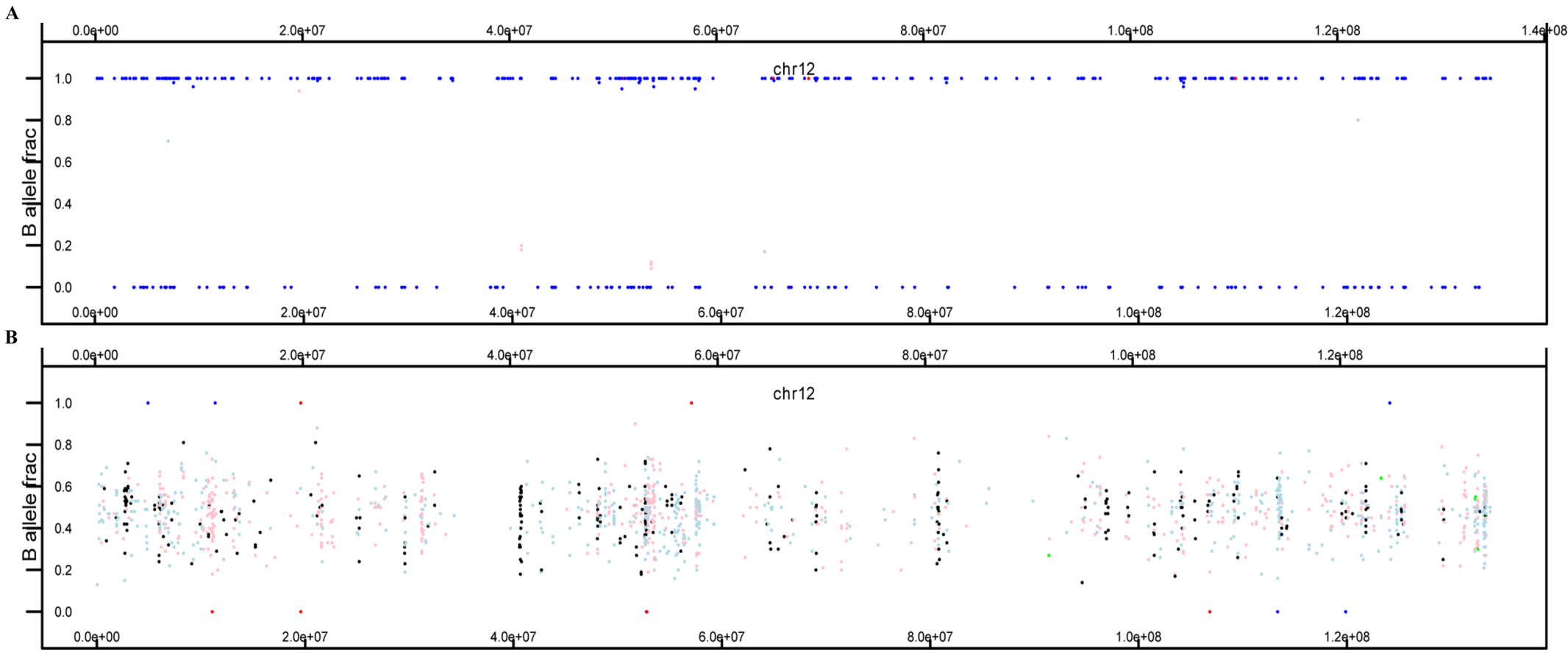
**
